# ASP-based method for the enumeration of attractors in non-deterministic synchronous and asynchronous multi-valued networks

**DOI:** 10.1186/s13015-017-0111-2

**Published:** 2017-08-15

**Authors:** Emna Ben Abdallah, Maxime Folschette, Olivier Roux, Morgan Magnin

**Affiliations:** 1 École Centrale de Nantes, LS2N UMR CNRS 6004, 1 rue de la Noë, 44321 Nantes, France; 2 Université de Nantes, LS2N UMR CNRS 6004, 1 rue de la Noë, 44321 Nantes, France; 3 Université Nice Sophia Antipolis, I3S UMR CNRS 7271, 2000, route des Lucioles, 06900 Nice, France; 4 National Institute of Informatics, 2-1-2, Hitotsubashi, Chiyoda-ku, Tokyo, 101-8430 Japan

**Keywords:** Biological regulatory network, Multiple-valued networks, Attractors, Steady states, Cycles, Answer set programming

## Abstract

**Background:**

This paper addresses the problem of finding attractors in biological regulatory networks. We focus here on non-deterministic synchronous and asynchronous multi-valued networks, modeled using automata networks (AN). AN is a general and well-suited formalism to study complex interactions between different components (genes, proteins,...). An attractor is a minimal trap domain, that is, a part of the state-transition graph that cannot be escaped. Such structures are terminal components of the dynamics and take the form of steady states (singleton) or complex compositions of cycles (non-singleton). Studying the effect of a disease or a mutation on an organism requires finding the attractors in the model to understand the long-term behaviors.

**Results:**

We present a computational logical method based on answer set programming (ASP) to identify all attractors. Performed without any network reduction, the method can be applied on any dynamical semantics. In this paper, we present the two most widespread non-deterministic semantics: the asynchronous and the synchronous updating modes. The logical approach goes through a complete enumeration of the states of the network in order to find the attractors without the necessity to construct the whole state-transition graph. We realize extensive computational experiments which show good performance and fit the expected theoretical results in the literature.

**Conclusion:**

The originality of our approach lies on the exhaustive enumeration of all possible (sets of) states verifying the properties of an attractor thanks to the use of ASP. Our method is applied to non-deterministic semantics in two different schemes (asynchronous and synchronous). The merits of our methods are illustrated by applying them to biological examples of various sizes and comparing the results with some existing approaches. It turns out that our approach succeeds to exhaustively enumerate on a desktop computer, in a large model (100 components), all existing attractors up to a given size (20 states). This size is only limited by memory and computation time.

## Background

In the last decades, the emergence of a wide range of new technologies have made it possible to produce a massive amount of biological data (genomics, proteomics...). This leads to considerable developments in systems biology which takes profit from this data. In order to understand the nature of a cellular function or more broadly a living biological system (healthy or diseased), it is indeed essential to study not only the individual properties of cellular components, but also their interactions. The behavior and functionalities of the cells emerge from such networks of interactions.

Considering this paradigm, the long-term behavior of regulatory networks dynamics is of specific interest [1]. Indeed, at any moment, a system may fall into a trap domain, which is a part of its dynamics that cannot be escaped. While evolving, the system may eventually fall into a new and smaller trap domain, which reduces its possible future behaviors (making previous states no longer reachable). This phenomenon depends on biological disruptions or other complex phenomena. Such outline has been interpreted as distinct responses of the organism, such as differentiating into distinct cell types in multicellular organisms [2].

Moreover, when refining a model of a living system, one way to remove inconsistencies or to predict missing information in biological models consists in comparing the attractors of the model with the experimentally observed long-term behavior. For instance, the model of the cellular development of *Drosophila melanogaster*, was described using Boolean networks and their attractors [3, 4].

Various kinds of mathematical models have been proposed for the modeling of biological regulatory networks (BRNs). These models include neural networks, differential equations, Petri nets, Boolean networks (BN) as proposed by Kauffman [5], probabilistic Boolean networks, and other multi-valued models such synchronous/asynchronous automata networks (AN). In this paper, we use the AN formalism [6, 7] to model BRNs. ANs especially encompass the framework of René Thomas [8].

Qualitative frameworks have received substantial attention, because of their capacity to capture the switching behavior of genetic or biological processes, and therefore, the study of their long-term behavior. This explains our choice of a qualitative representation for the identification of trap domains. In such a qualitative framework, a minimal trap domain can take two different forms: it can be either a steady state, which is one state from which the system does not evolve anymore, called also a fixed point; or an attractor, which is a minimal set of states that loops indefinitely and cannot be escaped.

The computational problem of finding all attractors in a BRN is difficult. Even the simpler problem of deciding whether the system has a fixed point, which can be seen as the smallest kind of attractor, is NP-hard [9]. Based on this, many studies have proven that computing attractors in BRNs is also a NP-hard problem [10, 11]. Although some methods exist with a lesser complexity, consisting for instance in randomly selecting an initial state and following a long enough trajectory, hoping to eventually finding an attractor, they are not exhaustive and may miss some (hard to reach) attractors.

Therefore, in the absence of more efficient exhaustive methods, it is still relevant to develop an approach to resolve the original NP-hard problem of attractors identification. Such an approach consists in exhaustively examine all possible states of a network, along with all possible paths from each of these states. Obviously, this brute force method is very time and memory consuming: $2^n$ initial states have to be considered for a Boolean model with *n* nodes; and multi-valued networks raise this value even more. Furthermore, a sufficient number of computations have to be performed to ensure that all trajectories have been explored and all attractors are found. This high complexity justifies the use of a tool able to tackle such hard problems.

The simplest way to detect attractors is to enumerate all the possible states and to run simulation from each one until an attractor is reached [12]. This method ensures that all attractors are detected but it has an exponential time complexity, therefore its applicability is highly restricted by the network size.

Regarding BNs only, algorithms for detecting attractors have been extensively studied in the literature. Irons [13] proposes to analyze partial states in order to discard potential attractors more efficiently. This method improves the efficiency from exponential time to polynomial time for a subset of biological Boolean models that is highly dependent on the topology (indegree, outdegree, update functions) of the underlying network. Another method, called GenYsis [14], starts from one (randomly selected) initial state and detects attractors by computing the successor and predecessor states of this initial state. It works well for small BNs, but becomes inefficient for large BNs.

More generally, the efficiency and scalability of attractor detection techniques are further improved with the integration of two techniques. This first is based on binary decision diagrams (BDD), a compact data structure for representing Boolean functions. In a recent work [15], algorithms have been based on the reduced-order BDD (ROBDD) data structure, which further speeds up the computation time of attractor detection. These BDD-based solutions only work for BRNs of a hundred of nodes and also suffer from the infamous state explosion problem, as the size of the BDD depends both on the regulatory functions and the number of nodes in the BRN. The other technique consists in representing the attractor enumeration problem as a satisfiability (SAT) problem such as in [16]. The main idea is inspired by SAT-based bounded model-checking: the transition relation of the BRN is unfolded into a bounded number of steps in order to construct a propositional formula which encodes attractors and which is then solved by a SAT solver. In every step, a new variable is required to represent a state of a node in the BRN. It is clear that the efficiency of these algorithms largely depends on the number of unfolding steps and the number of nodes in the BRN.

In [17], the authors separated the rules that describe the network (the nodes and their interactions: activation or inhibition) from the rules that define its dynamics (for instance: a gene will be activated in the next state if all its activators are active or when at least one of its activators is active at the current state). This allows to obtain more flexible simulations, and the authors also chose to use the declarative paradigm answer set programming (ASP) [18] in order to have more liberty in the expression of evolution rules. They illustrated that specifying large networks with rather complicated behaviors becomes cumbersome and error prone in paradigms like SAT, whereas this is much less the case in a declarative approach such as theirs.

Our goal in this paper is to develop exhaustive methods to analyze a BRN modeled in AN. We address two kinds of issues: finding all possible steady states of a BRN and enumerating all attractors of a given size $n \ge 2$. We focus on two widespread non-deterministic update schemes (synchronous and asynchronous) and use ASP to solve these aforementioned issues. Although this approach is not new (see above), the use of ASP can still be considered innovative in the field of dynamic properties analysis and our aim here is to assess its computational potential.

Nevertheless, the originality of our contribution is to consider AN models: this formalism does not restrict entities to have Boolean expression levels (active/inactive) as they can have multi-valued ones. Complex interactions are modeled in an AN as automata transitions instead of generic influences. This expressiveness allows to represent a wide range of dynamical models with the AN framework, and the particular form of its local transitions can be well handled in ASP. Finally, this framework allows to represent non-deterministic synchronous models, contrary to previous works focusing on asynchronous or deterministic synchronous models.

We previously introduced some rough ideas of this approach in [19]. In the present paper, we have extended this work by focusing on AN models, that are more expressive than the previous process hitting framework [20]. We give a more detailed state-of-the-art and a more in-depth formalization of the problems tackled and show the merits of our approach on a case study and various benchmarks.

This paper is organized as follows. "Automata networks" presents the main definitions related to the AN and the particular constructs that we will seek: fixed points and attractors. "Answer set programming" briefly presents the ASP framework necessary to understand the encoding part. Section "Fixed points enumeration" details the part of our method that allows to present an AN model using ASP rules and find all the fixed points in such a model. Then, "Length* n* attractors enumeration" explains how to enumerate all attractors in a model still using ASP. In "Results" we give benchmarks of our methods on several models of different sizes (up to 100 components). Finally, “Conclusion and future direction” concludes and gives some perspectives to this work.

## Preliminary definitions

### Automata networks

**Fig. 1:**
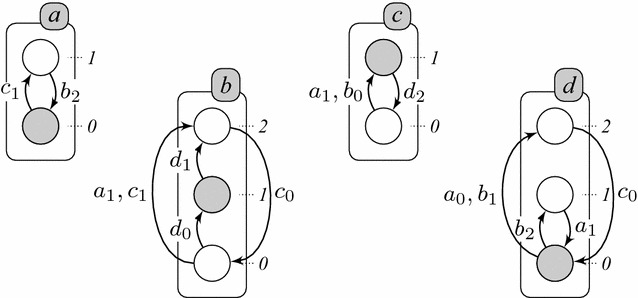
An example of an AN model with 4 automata: *a*, *b*, *c* and *d*.* Each box* represents an automaton (modeling a biological component),* circles* represent their local states (corresponding to their discrete expression levels) and the local transitions are represented by* arrows* labeled by their necessary conditions (consisting of a set of local states from other automata). The automata *a* and *c* are either at level 0 or 1, and *b* and *d* have 3 levels (0, 1 and 2). The* grayed* local states stand for the network state $\langle a_0, b_1, c_1, d_0 \rangle$

Definition 1 introduces the formalism of automata networks (AN) [6] (see Fig. 1) which allows to model a finite number of discrete levels, called local states, into several automata. A local state is denoted $a_i$, where *a* is the name of the automaton, corresponding usually to a biological component, and *i* is a level identifier within *a*. At any time, exactly one local state of each automaton is active, modeling the current level of activity or the internal state of the automaton. The set of all active local states is called the global state of the network.

The possible local evolutions inside an automaton are defined by local transitions. A local transition is a triple noted $a_i \overset{\ell }{\rightarrow } a_j$ and is responsible, inside a given automaton *a*, for the change of the active local state ($a_i$) to another local state ($a_j$), conditioned by the presence of a set $\ell$ of local states belonging to other automata and that must be active in the current global state. Such a local transition is playable if and only if $a_i$ and all local states in the set $\ell$ are active. Thus, it can be read as “all the local states in $\ell$ can cooperate to change the active local state of *a* by making it switch from $a_i$ to $a_j$”. It is required that $a_i$ and $a_j$ are two different local states in automaton *a*, and that $\ell$ contains no local state of automaton *a*. We also note that $\ell$ should contain at most one local state per automaton, otherwise the local transition is unplayable; $\ell$ can also be empty.

#### **Definition 1**

(*Automata network*) An *Automata network* is a triple $(\Sigma ,\mathcal {S},\mathcal {T})$ where:

- $\Sigma = \{a, b,\ldots \}$ is the finite set of *automata* identifiers;
- For each $a \in \Sigma$, $\mathcal {S}_a = \{a_i,\ldots ,a_j\}$ is the finite set of *local states* of automaton *a*; $\mathcal {S}= \prod _{a \in \Sigma }\mathcal {S}_a$ is the finite set of *global states*; $\user2{LS} =  \cup _{{a \in \Sigma }} {\mathcal{S}}_{a}$ denotes the set of all the local states.
- For each $a \in \Sigma$, $\mathcal {T}_a = \{ a_i \overset{\ell }{\rightarrow } a_j \in \mathcal {S}_a \times \wp (\user2{LS} \setminus \mathcal {S}_a) \times \mathcal {S}_a \mid a_i \ne a_j \}$ is the set of *local transitions* on automaton *a*; $\mathcal {T}= \bigcup _{a \in \Sigma } \mathcal {T}_a$ is the set of all local transitions in the model.

For a given local transition $\tau = a_i \overset{\ell }{\rightarrow } a_j$, $a_i$ is called the origin or $\tau$, $\ell$ the condition and $a_j$ the destination, and they are respectively noted $\mathsf {ori}(\tau )$, $\mathsf {cond}(\tau )$ and $\mathsf {dest}(\tau )$.

#### *Example 1*

Figure 1 represents an AN $(\Sigma , \mathcal {S}, \mathcal {T})$ with 4 automata (among which two contain 2 local states and the two others contain 3 local states) and 12 local transitions:

- $$\Sigma = \{a, b, c, d\}$$,
- $$\mathcal {S}_a = \{a_0, a_1\}$$, $$\mathcal {S}_b = \{b_0, b_1, b_2\}$$, $$\mathcal {S}_c = \{c_0, c_1\}$$, $$\mathcal {S}_d = \{d_0, d_1, d_2\} $$,
- $$\mathcal {T}= \{ \begin{array}[t]{ll} a_0 \overset{\{c_1\}}{\longrightarrow } a_1, a_1 \overset{\{b_2\}}{\longrightarrow } a_0, &{} b_0 \overset{\{d_0\}}{\longrightarrow } b_1, b_0 \overset{\{a_1, c_1\}}{\longrightarrow } b_2, b_1 \overset{\{d_1\}}{\longrightarrow } b_2, b_2 \overset{\{c_0\}}{\longrightarrow } b_0, \\ c_0 \overset{\{a_1, b_0\}}{\longrightarrow } c_1, c_1 \overset{\{d_2\}}{\longrightarrow } c_0, &{} d_0 \overset{\{b_2\}}{\longrightarrow } d_1, d_0 \overset{\{a_0, b_1\}}{\longrightarrow } d_2, d_1 \overset{\{a_1\}}{\longrightarrow } d_0, d_2 \overset{\{c_0\}}{\longrightarrow } d_0 \}\text {.} \end{array}$$

The local transitions given in Definition 1 thus define concurrent interactions between automata. They are used to define the general dynamics of the network, that is, the possible global transitions between global states, according to a given update scheme. In the following, we will only focus on the (purely) asynchronous and (purely) synchronous update schemes, which are the most widespread in the literature. The choice of such an update scheme mainly depends on the considered biological phenomena modeled and the mathematical abstractions chosen by the modeler.

### Update schemes and dynamics of automata networks

As explained in the previous section, a global state of an AN is a set of local states of automata, containing exactly one local state of each automaton. In the following, we give some notations related to global states, then we define the global dynamics of an AN.

The active local state of a given automaton $a \in \Sigma$ in a global state $\zeta \in \mathcal {S}$ is noted ${\zeta [a]}$. For any given local state $a_i \in {\mathbf{LS}}$, we also note: $a_i \in \zeta$ if and only if ${\zeta [a]} = a_i$, which means that the biological component *a* is in the discrete expression level labeled *i* within state $\zeta$. For a given set of local states $X \subseteq \mathbf {LS}$, we extend this notation to $X \subseteq \zeta$ if and only if $\forall a_i \in X, a_i \in \zeta$, meaning that all local states of *X* are active in $\zeta$.

Furthermore, for any given local state $a_i \in \mathbf {LS}$, $\zeta \Cap a_i$ represents the global state that is identical to $\zeta$, except for the local state of *a* which is substituted with $a_i$: ${(\zeta \Cap a_i)[a]} = a_i \wedge \forall b \in \Sigma{\setminus}\{ a \}, {(\zeta \Cap a_i)[b]} = {\zeta [b]}$. We generalize this notation to a set of local states $X \subseteq \mathbf {LS}$ containing at most one local state per automaton, that is, $\forall a \in \Sigma , |X \cap \mathcal {S}_a| \le 1$ where $|S|$ is the number of elements in set *S*; in this case, $\zeta \Cap X$ is the global state $\zeta$ where the local state of each automaton has been replaced by the local state of the same automaton in *X*, if there exists: $\forall a \in \Sigma , (X \cap \mathcal {S}_a = \{ a_i \} \Rightarrow {(\zeta \Cap X)[a]} = a_i) \wedge (X \cap \mathcal {S}_a = \emptyset \Rightarrow {(\zeta \Cap X)[a]} = {\zeta [a]})$.

In Definition 2 we formalize the notion of playability of a local transition which was informally presented in the previous section. Playable local transitions are not necessarily used as such, but combined depending on the chosen update scheme, which is the subject of the rest of the section.

#### **Definition 2**

(*Playable local transitions*) Let $\mathcal {AN}= (\Sigma ,\mathcal {S},\mathcal {T})$ be an automata network and $\zeta \in \mathcal {S}$ a global state. The set of playable local transitions in $\zeta$ is called $P_\zeta$ and defined by: $P_\zeta = \{ a_i \overset{\ell }{\rightarrow } a_j \in \mathcal {T}\mid \ell \subseteq \zeta \wedge a_i \in \zeta \}$.

The dynamics of the AN is a composition of global transitions between global states, that consist in applying a set of local transitions. Such sets are different depending on the chosen update scheme. In the following, we give the definition of the asynchronous and synchronous update schemes by characterizing the sets of local transitions that can be “played” as global transitions. The asynchronous update sets (Definition 3) are made of exactly one playable local transition; thus, a global asynchronous transition changes the local state of exactly one automaton. On the other hand, the synchronous update sets (Definition 4) consist of exactly one playable local transition for each automaton (except the automata where no local transition is playable); in other words, a global synchronous transition changes the local state of all automata that can evolve at a time. Empty update sets are not allowed for both update schemes. In the definitions below, we willingly mix the notions of “update scheme” and “update set”, which are equivalent here.

#### **Definition 3**

(*Asynchronous update scheme*) Let $\mathcal {AN}= (\Sigma , \mathcal {S}, \mathcal {T})$ be an automata network and $\zeta \in \mathcal {S}$ a global state. The set of global transitions playable in $\zeta$ for the asynchronous update scheme is given by:

$$\begin{aligned} U^{\mathsf {asyn}}(\zeta ) = \{ \{ a_i \overset{\ell }{\rightarrow } a_j\} \mid a_i \overset{\ell }{\rightarrow } a_j\in P_\zeta \}. \end{aligned}$$

#### **Definition 4**

(*Synchronous update scheme*) Let $\mathcal {AN}= (\Sigma ,\mathcal {S},\mathcal {T})$ be an automata network and $\zeta \in \mathcal {S}$ a global state. The set of global transitions playable in $\zeta$ for the synchronous update scheme is given by:

$$\begin{aligned} U^{\mathsf {syn}}(\zeta )&= \{ u \subseteq \mathcal {T}\mid u \ne \emptyset \wedge \forall a \in \Sigma , (P_\zeta \cap \mathcal {T}_a = \emptyset \Rightarrow u \cap \mathcal {T}_a = \emptyset ) \wedge \\& \quad(P_\zeta \cap \mathcal {T}_a \ne \emptyset \Rightarrow |u \cap \mathcal {T}_a| = 1) \}. \end{aligned}$$

Once an update scheme has been chosen, it is possible to compute the corresponding dynamics of a given AN. Thus, in the following, when it is not ambiguous and when results apply to both of them, we will denote by $U^{}$ a chosen update scheme among $U^{\mathsf {asyn}}$ and $U^{\mathsf {syn}}$. Definition 5 formalizes the notion of a global transition depending on a chosen update scheme $U^{}$.

#### **Definition 5**

(*Global transition*) Let $\mathcal {AN}= (\Sigma ,\mathcal {S},\mathcal {T})$ be an automata network, $\zeta _1, \zeta _2 \in \mathcal {S}$ two states and $U^{}$ an update scheme (i.e., $U^{}\in \{ U^{\mathsf {asyn}}, U^{\mathsf {syn}}\}$). The *global transition* relation between two states $\zeta _1$ and $\zeta _2$ for the update scheme represented by $U^{}$, noted $\zeta _1 \rightarrow _{U^{}} \zeta _2$, is defined by:

$$\begin{aligned} \zeta _1 \rightarrow _{U^{}} \zeta _2 \Longleftrightarrow \exists u \in U^{}(\zeta _1), \quad \zeta _2 = \zeta _1 \Cap \{ \mathsf {dest}(\tau ) \in \mathbf LS \mid \tau \in u \}. \end{aligned}$$

The state $\zeta _2$ is called a *successor* of $\zeta _1$.

We note that in a deterministic dynamics, each state has only one successor. However, in case of non-deterministic dynamics, such as the asynchronous and synchronous update schemes of this paper, each state may have several possible successors.

#### *Example 2*

**Fig. 2:**
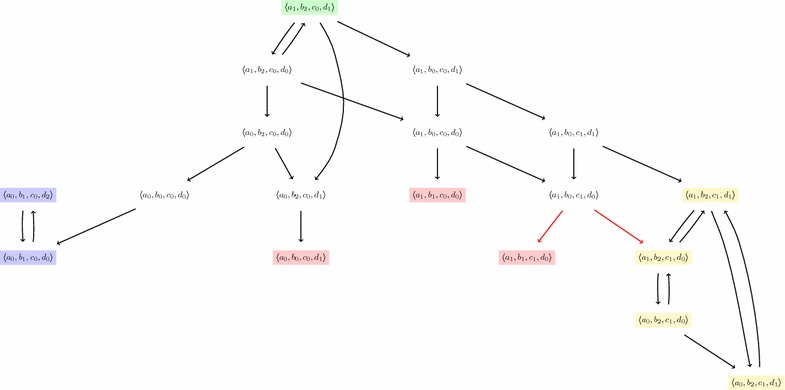
A part of the state-transition graph of the AN given in Fig. 1 for the *asynchronous* update scheme, computed from the initial state: $\langle a_1, b_2, c_0, d_1 \rangle$ until reaching attractors. We can observe three fixed points: $\langle a_1, b_1, c_1, d_0 \rangle$, $\langle a_1, b_1, c_0, d_0 \rangle$ and $\langle a_0, b_0, c_0, d_1 \rangle$; an attractor of size 2: $\{ \langle a_0, b_1, c_0, d_0 \rangle , \langle a_0, b_1, c_0, d_2 \rangle \}$ (in* blue*) and an attractor of size 4: $\{ \langle a_1, b_2, c_1, d_1 \rangle ,\langle a_0, b_2, c_1, d_1 \rangle ,\langle a_0, b_2, c_1, d_0 \rangle ,\langle a_1, b_2, c_1, d_0 \rangle \}$ (in* yellow*)

**Fig. 3:**
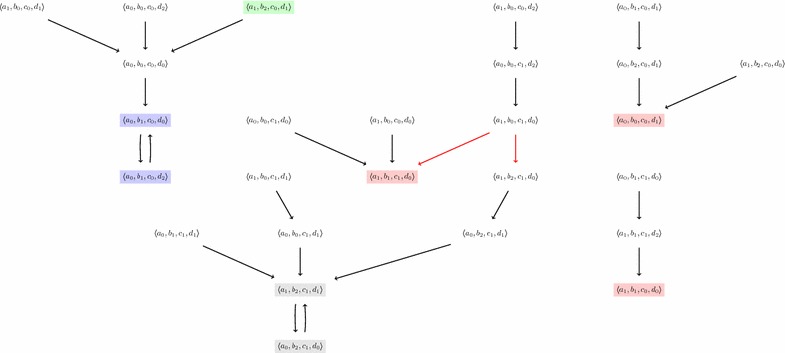
A part of the state-transition graph of the AN given in Fig. 1 for the *synchronous* update scheme, computed from several initial states, such as $\langle a_1, b_2, c_0, d_1 \rangle$, until reaching attractors. It features non-deterministic global transitions, depicted by the* two red arrows*. We can observe the same three fixed points than for the asynchronous update scheme of Fig. 2, but instead two attractors of size 2: $\{ \langle a_0, b_1, c_0, d_0 \rangle , \langle a_0, b_1, c_0, d_2 \rangle \}$ (in* blue*) and $\{ \langle a_1, b_2, c_1, d_1 \rangle , \langle a_0, b_2, c_1, d_0 \rangle \}$ (in* gray*)

Figures 2 and 3 illustrate respectively the asynchronous and synchronous update schemes on the model of Fig. 1. Each global transition is depicted by an arrow between two global states. Only an interesting subset of the whole dynamics is depicted in both figures.

At this point, it is important to remind that the empty set never belongs to the update schemes defined above: $\forall \zeta \in \mathcal {S}, \emptyset \notin U^{\mathsf {asyn}}(\zeta ) \wedge \emptyset \notin U^{\mathsf {syn}}(\zeta )$. The consequence on the dynamics is that a global state can never be its own successor. In other words, even when no local transition can be played in a given global state (i.e., $P_\zeta = \emptyset$), we do not add a “self-transition” on this state. Instead, this state has no successors and is called a fixed point, as defined later in this section.

Definition 6 explains what are in-conflict local transitions, which are interesting in the scope of the synchronous update scheme. Two local transitions are in-conflict if they belong to the same automaton and produce some non-determinism inside this automaton. Such phenomenon arises when both local transitions have the same origin and compatible conditions, but their destinations are different; or, in other words, there exists a global state in which they are both playable. In such a case, they allow the automaton to evolve in two different possible local states from the same active local state, thus producing a non-deterministic behavior. This definition will be used in the discussion of the next section and in "Length* n* attractors enumeration".

#### **Definition 6**

(*In-conflict local transitions*) Let $\mathcal {AN}= (\Sigma ,\mathcal {S},\mathcal {T})$ be an automata network, $a \in \Sigma$ an automaton and $\tau _1, \tau _2 \in \mathcal {T}_a$ two local transitions in this automaton. $\tau _1$ and $\tau _2$ are said *in-conflict* if and only if:

$$\begin{aligned} \mathsf {ori}(\tau _1) = \mathsf {ori}(\tau _2) \wedge \mathsf {dest}(\tau _1) \ne \mathsf {dest}(\tau _2) \wedge \exists \zeta \in \mathcal {S}\quad \text{ such that } \tau _1 \in P_\zeta \wedge \tau _2 \in P_\zeta . \end{aligned}$$

Finally, Definition 7 introduces the notions of path and trace which are used to characterize a set of successive global states with respect to a global transition relation. Paths are useful for the characterization of attractors that are the topic of this work. The trace is the set of all global states traversed by a given path (thus disregarding the order in which they are visited). We note that a path is a sequence and a trace is a set.

#### **Definition 7**

(*Path and trace*) Let $\mathcal {AN}= (\Sigma ,\mathcal {S},\mathcal {T})$ be an automata network, $U^{}$ an update scheme and $n \in \mathbb {N}\setminus \{ 0 \}$ a strictly positive integer. A sequence $H = ( H_i )_{i \in \llbracket 0; n \rrbracket } \in \mathcal {S}^{n+1}$ of global states is a *path of length n* if and only if: $\forall i \in \llbracket 0; n-1 \rrbracket , H_i \rightarrow _{U^{}} H_{i+1}$. *H* is said to *start from* a given global state $\zeta \in \mathcal {S}$ if and only if: $H_0 = \zeta$. Finally, the *trace* related to such a path is the set of the global states that have been visited: $\mathsf {trace}(H) = \{ H_j \in \mathcal {S}\mid j \in \llbracket 0; n \rrbracket \}$.

In the following, when we define a path *H* of length *n*, we use the notation $H_i$ to denote the *i*th element in the sequence *H*, with $i \in \llbracket 0; n \rrbracket$. We also use the notation $|H| = n$ to denote the length of a path *H*, allowing to write: $H_{|H|}$ to refer to its last element. We also recall that a path of length *n* models the succession of *n* global transitions, and thus features up to* n* + 1 states (some states may be visited more than once).

#### *Example 3*

The following sequence is a path of length 6 for the asynchronous update scheme:

$$\begin{aligned} H&= ( \langle a_1, b_2, c_1, d_1 \rangle ; \langle a_0, b_2, c_1, d_1 \rangle ; \langle a_1, b_2, c_1, d_1 \rangle ;\\ &\quad  \langle a_1, b_2, c_1, d_0 \rangle ; \langle a_0, b_2, c_1, d_0 \rangle ; \langle a_0, b_2, c_1, d_1 \rangle ;\\ &\quad  \langle a_1, b_2, c_1, d_1 \rangle ) \end{aligned}$$

We have: $\mathsf {trace}(H) = \{ \langle a_1, b_2, c_1, d_1 \rangle , \langle a_0, b_2, c_1, d_1 \rangle , \langle a_1, b_2, c_1, d_0 \rangle , \langle a_0, b_2, c_1, d_0 \rangle \}$ and: $|\mathsf {trace}(H)| = 4$. We note that $H_0 = H_2 = H_6$ and $H_1 = H_5$.

When there is one or several repetitions in a given path of length *n* (i.e., if a state is visited more than once), its trace is then of size strictly lesser than* n* + 1. More precisely, one can compute the size of the trace corresponding to a given path by subtracting the number of repetitions in that path (Lemma 1). For this, we formalize in Definition 8 the notion of repetitions in a path, that is, the global states that are featured several times, designated by their indexes.

#### **Definition 8**

(*Repetitions in a path*) Let $\mathcal {AN}= (\Sigma ,\mathcal {S},\mathcal {T})$ be an automata network, $n \in \mathbb {N}{\setminus}\{0\}$ a strictly positive integer and *H* a path of length *n*. The set of *repetitions* in *H* is given by:

$$\begin{aligned} \mathsf {sr}(H) = \{ i \in \llbracket 1; n \rrbracket \mid \exists j \in \llbracket 0; i-1 \rrbracket , H_j = H_i \}. \end{aligned}$$

#### **Lemma 1**

(Size of a trace)* Let* *H* *be a path of length* *n*.* The number of elements in its trace is given by:*

$$\begin{aligned} |\mathsf {trace}{(H)}| = n + 1 - |\mathsf {sr}(H)|. \end{aligned}$$

#### *Proof of Lemma 1*

By definition of a set, and knowing that $|\mathsf {sr}(H)|$ counts the number of states that exist elsewhere in *H* with a lesser index. $\square$

We note that if there is no repetition in a path of length *n* ($\mathsf {sr}(H)=\emptyset \Rightarrow |\mathsf {sr}(H)|=0$), then the number of visited states is exactly: $|\mathsf {trace}{(H)}| = n+1$.

#### *Example 4*

We can check Lemma 1 on the path *H* given in Example 3. Indeed, $\langle a_1, b_2, c_1, d_1 \rangle$ is featured 3 times at $H_0$, $H_2$ and $H_6$. Then, according to the Definition 8, this state is repeated twice at $H_2$ and $H_6$ because the first visit of this state is not computed in $\mathsf {sr}(H)$. In addition, the state $\langle a_0, b_2, c_1, d_1 \rangle$ is featured twice in this path, at $H_1$ and $H_5$, therefore it is considered as repeated once at $H_5$. Thus, $\mathsf {sr}(H)=\{2,6,5\}$, $|\mathsf {sr}(H)|=3$ and $|\mathsf {trace}(H)| = 6 + 1 - 3 = 4$.

### Determinism and non-determinism of the update schemes

In the general case, in multi-valued networks, both the asynchronous and synchronous update schemes are non-deterministic, which means that a global state can have several successors.

In the case of the asynchronous update scheme, the non-determinism may come from in-conflict local transitions, but it actually mainly comes from the fact that exactly one local transition is taken into account for each global transition (see Definition 3). Thus, for a given state $\zeta \in \mathcal {S}$, as soon as $|P_\zeta | > 1$, several successors may exist. In the model of Fig. 1, for example, the global state $\langle a_1, b_2, c_0, d_1 \rangle$ (in green on Fig. 2) has three successors: $\langle a_1, b_2, c_0, d_1 \rangle \rightarrow _{U^{\mathsf {asyn}}} \langle a_0, b_2, c_0, d_1 \rangle$, $\langle a_1, b_2, c_0, d_1 \rangle \rightarrow _{U^{\mathsf {asyn}}} \langle a_1, b_0, c_0, d_1 \rangle$ and $\langle a_1, b_2, c_0, d_1 \rangle \rightarrow _{U^{\mathsf {asyn}}} \langle a_1, b_2, c_0, d_0 \rangle$.

In the case of the synchronous update scheme (see Definition 4), however, the non-determinism on the global scale is only generated by in-conflict local transitions (see Definition 6), that is, local transitions that create non-determinism inside an automaton. For example, the model of Fig. 1 features two local transitions $b_0 \overset{\{d_0\}}{\longrightarrow } b_1$ and $b_0 \overset{\{a_1, c_1\}}{\longrightarrow } b_2$ that can produce the two following global transitions from the same state (depicted by red arrows on Fig. 3): $\langle a_1, b_0, c_1, d_0 \rangle \rightarrow _{U^{\mathsf {syn}}} \langle a_1, b_1, c_1, d_0 \rangle$ and $\langle a_1, b_0, c_1, d_0 \rangle \rightarrow _{U^{\mathsf {syn}}} \langle a_1, b_2, c_1, d_0 \rangle$. Note that for this particular case, these transitions also exist for the asynchronous scheme (also depicted by red arrows on Fig. 2).

Therefore, it is noteworthy that if every automaton contains only two local states (such a network is often called “Boolean”) then the synchronous update scheme becomes completely deterministic. Indeed, it is not possible to find in-conflict local transitions anymore because for each possible origin of a local transition, there can be only one destination (due to the fact that the origin and destination of a local transition must be different). This observation can speed up the computations in this particular case.

### Fixed points and attractors in automata networks

Studying the dynamics of biological networks was the focus of many works, explaining the diversity of existing frameworks dedicated to modeling and the different methods developed in order to identify some patterns, such as attractors  [9, 11, 17, 21, 22]. In this paper we focus on several sub-problems related to this: we seek to identify the steady states and the attractors of a given network. The steady states and the attractors are the two long-term structures in which any dynamics eventually falls into. Indeed, they consist in terminal (sets of) global states that cannot be escaped, and in which the dynamics always ends.

In the following, we consider a BRN modeled in AN $(\Sigma ,\mathcal {S},\mathcal {T})$, and we formally define these dynamical properties. We note that since the AN formalism encompasses Thomas modeling [8], all our results can be applied to the models described by this formalism, as well as any other framework that can be described in AN (such as Boolean networks, Biocham [23]...).

A fixed point is a global state which has no successor, as given in Definition 9. Such global states have a particular interest as they denote conditions in which the model stays indefinitely. The existence of several of these states denotes a multistability, and possible bifurcations in the dynamics [1].

#### **Definition 9**

(*Fixed point*) Let $\mathcal {AN}= (\Sigma ,\mathcal {S},\mathcal {T})$ be an automata network, and $U^{}$ be an update scheme ($U^{}\in \{ U^{\mathsf {asyn}}, U^{\mathsf {syn}}\}$). A global state $\zeta \in \mathcal {S}$ is called a *fixed point* (or equivalently *steady state*) if and only if no global transition can be played in this state:

$$\begin{aligned} U^{}(\zeta ) = \emptyset . \end{aligned}$$

It is notable that the set of fixed points of a model (that is, the set of states with no successor) is the same in both update schemes asynchronous and synchronous update [24, 25]: $\forall \zeta \in \mathcal {S}, U^{\mathsf {asyn}}(\zeta ) = \emptyset \Longleftrightarrow U^{\mathsf {syn}}(\zeta ) = \emptyset .$

#### *Example 5*

The state-transition graphs of Figs. 2 and 3 depict three fixed points colored in red: $\langle a_1, b_1, c_1, d_0 \rangle$, $\langle a_1, b_1, c_0, d_0 \rangle$ and $\langle a_0, b_0, c_0, d_1 \rangle$. Visually, they can be easily recognized because they have no outgoing arrow (meaning that they have no successors). Although these figures do not represent the whole dynamics, but they allow to check that in both update schemes the fixed points are the same, at least on this subset of the overall behavior.

Another complementary dynamical pattern consists in the notion of non-unitary trap domain (Definition 10), which is a (non-singleton) set of states that the dynamics cannot escape, and thus in which the system indefinitely remains. In this work, we focus more precisely on (non-singleton) attractors (Definition 11), that are cyclic and minimal trap domains in terms of sets inclusion. In order to characterize such attractors, we use the notion of cycle (Definition 12), which is a looping path. Indeed, a cycle is a strongly connected component (Lemma 2), allowing us to give an alternative definition for an attractor (Lemma 3). Formally speaking, fixed points can be considered as attractors of size 1. However, in the scope of this paper and for the sake of clarity, we call “attractors” only non-unitary attractors, that is, only sets containing at least two states. This is justified by the very different approaches developed for fixed points and attractors in the next sections.

#### **Definition 10**

(*Trap domain*) Let $\mathcal {AN}= (\Sigma ,\mathcal {S},\mathcal {T})$ be an automata network and $U^{}$ an update scheme. A set of global states $\mathbf {T}$, with $|\mathbf {T}| \ge 2$, is called a *trap domain* (regarding a scheme $U^{}$) if and only if the successors of each of its elements are also in $\mathbf {T}$:

$$\begin{aligned} \forall \zeta _1 \in \mathbf {T} \wedge \forall \zeta _2 \in \mathcal {S}\text { if } \zeta _1 \rightarrow _{U^{}} \zeta _2 \quad \text{ then } \zeta _2 \in \mathbf {T}. \end{aligned}$$

#### **Definition 11**

(*Attractor*) Let $\mathcal {AN}= (\Sigma ,\mathcal {S},\mathcal {T})$ be an automata network and $U^{}$ an update scheme. A set of global states $\mathbf {A}$, with $|\mathbf {A}| \ge 2$, is called an *attractor* (regarding scheme $U^{}$) if and only if it is a minimal trap domain in terms of inclusion.

#### **Definition 12**

(*Cycle*) Let $\mathcal {AN}= (\Sigma ,\mathcal {S},\mathcal {T})$ be an automata network, $U^{}$ an update scheme and $\mathbf {C}$ a path of length *n* for this update scheme. $\mathbf {C}$ is called a *cycle* of length *n* (regarding a scheme $U^{}$) if and only if it loops back to its first state:

$$\begin{aligned} \mathbf {C}_n = \mathbf {C}_0. \end{aligned}$$

#### *Example 6*

The path *H* of length 6 given in Example 3 is a cycle because $H_0 = H_6$.

Lemma 2 states that the set of (traces of) cycles in a model is exactly the set of strongly connected components. Indeed, a cycle allows to “loop” between all states that it contains, and conversely, a cycle can be built from the states of any strongly connected component. This equivalence is used in the next lemma.

#### **Lemma 2**

(The traces of cycles are the SCCs)* The traces of the cycles are exactly the strongly connected components (with respect to the global transition relation)*.

#### Proof of Lemma 2

($\Rightarrow$) From any state of a cycle, it is possible to reach all the other states (by possibly cycling). Therefore, the trace of this cycle is a strongly connected component. ($\Leftarrow$) Let $\mathbf {S} = \{ \zeta _{i} \}_{i \in \llbracket 0; n \rrbracket }$ be a strongly connected component in which the elements are arbitrarily labeled. Because it is a strongly connected component, for all $i \in \llbracket 0; n \rrbracket$, there exists a path $H^i$ made of elements of $\mathbf {S}$ so that $H^i_0 = \zeta _i$ and $H^i_{|H^i|} = \zeta _{i+1}$ (or $H^n_{|H^n|} = \zeta _0$ for $i = n$). We create a path $\mathbf {C}$ by concatenation of all paths $H^0, H^1, \ldots , H^n$ by merging the first and last element of each successive path, which is identical: $\forall i \in \llbracket 0; n-1 \rrbracket , H^i_{|H^i|} = \zeta _{i+1} = H^{i+1}_0$. $\mathbf {C}$ is a cycle, because $\mathbf {C}_0 = H^0_0 = \zeta _0 = H^n_{|H^n|} = \mathbf {C}_{|\mathbf {C}|}$. Furthermore, $\forall i \in \llbracket 0; n \rrbracket , \zeta _i = H^i_0 \in \mathsf {trace}(\mathbf {C})$, thus $\mathbf {S} \subseteq \mathsf {trace}(\mathbf {C})$. Finally, only states from $\mathbf {S}$ have been used to build $\mathbf {C}$, thus $\mathsf {trace}(\mathbf {C}) \subseteq \mathbf {S}$. Therefore, $\mathsf {trace}(\mathbf {C}) = \mathbf {S}$. $\square$

In Definition 11, attractors are characterized in the classical way, that is, as minimal trap domains. However, we use an alternative characterization of attractors in this paper, due to the specifics of ASP: Lemma 3 states that an attractor can alternatively be defined as a trap domain that is also a cycle, and conversely. In other words, the minimality requirement is replaced by a cyclical requirement.

#### **Lemma 3**

(The attractors are the trap cycles)* The attractors are exactly the traces of cycles which are trap domains*.

#### Proof of Lemma 3

($\Rightarrow$) By definition, an attractor is a trap domain. It is also a strongly connected component, and thus, from Lemma 2, it is the trace of a cycle. ($\Leftarrow$) Let $\mathbf {C}$ be both a cycle and a trap domain. From Lemma 2, $\mathbf {C}$ is also a strongly connected component. Let us prove by contradiction that $\mathbf {C}$ is a minimal trap domain, by assuming that it is not minimal. This means that there exists a smaller trap domain $\mathbf {D} \subsetneq \mathbf {C}$. Let us consider $x \in \mathbf {D}$ and $y \in \mathbf {C} \setminus \mathbf {D}$. Because $\mathbf {D}$ is a trap domain, it exists no path between *x* and *y*; this is in contradiction with $\mathbf {C}$ being a strongly connected component (as both *x* and *y* belong to $\mathbf {C}$). Therefore, $\mathbf {C}$ is a minimal trap domain, and thus an attractor. $\square$

As explained before, Lemma 3 will beused in "Length* n* attractors enumeration". Indeed, directly searching for minimal trap domains would be too cumbersome; instead, we enumerate cycles of length *n* in the dynamics of the model and filter out those that are not trap domains. The remaining results are the attractors formed of cycles of length *n*. The previous lemma ensures the soundness and completeness of this search for a given value of *n*.

#### **Lemma 4**

(Characterization of non-attractors)* Let* $\mathbf {A} \subset \mathcal {S}$ *be a set of states. If* $\exists \zeta _1 \in \mathbf {A}$ *and* $\exists \zeta _2 \in \mathcal {S}\setminus \mathbf {A}$ *such that* $\zeta _1 \rightarrow _{U^{}} \zeta _2$ *then* $\mathbf {A}$ *is not an attractor*.

#### *Proof of Lemma 4*

By definition, $\mathbf {A}$ is not a trap domain (Definition 10) and thus it is not an attractor (Definition 11). $\square$

#### *Example 7*

The state-transition graphs of Figs. 2 and 3 feature different attractors:

- $\{ \langle a_0, b_1, c_0, d_0 \rangle , \langle a_0, b_1, c_0, d_2 \rangle \}$ is depicted in blue and appears in both figures. It is a cyclic attractor, because it contains exactly one cycle.
- $\{ \langle a_0, b_2, c_1, d_0 \rangle , \langle a_0, b_2, c_1, d_1 \rangle , \langle a_1, b_2, c_1, d_1 \rangle , \langle a_1, b_2, c_1, d_0 \rangle \}$ is only present for the asynchronous update scheme and is depicted in yellow on Fig. 2. It is a complex attractor, that is, a composition of several cycles.
- $\{ \langle a_1, b_2, c_1, d_1 \rangle , \langle a_0, b_2, c_1, d_0 \rangle \}$ is, on the contrary, only present for the synchronous update scheme and is depicted in gray on Fig. 3. It is also a cyclic attractor.

For each of these attractors, the reader can check that they can be characterized as cycles that are trap domains. For instance, the second attractor can be found by considering the following cycle:

$$\begin{aligned} \mathbf {A} = ( \langle a_0, b_2, c_1, d_0 \rangle ; \langle a_0, b_2, c_1, d_1 \rangle ; \langle a_1, b_2, c_1, d_1 \rangle ; \langle a_1, b_2, c_1, d_0 \rangle ; \langle a_0, b_2, c_1, d_0 \rangle ) \end{aligned}$$

and checking that its trace is a trap domain (which is visually confirmed in Fig. 2 by the absence of outgoing arrows from any of the yellow states).

On the other hand, the following cycle is not an attractor:

$$\begin{aligned} \mathbf {C} = ( \langle a_1, b_2, c_0, d_1 \rangle ; \langle a_1, b_2, c_0, d_0 \rangle ; \langle a_1, b_2, c_0, d_1 \rangle ). \end{aligned}$$

Indeed, although it is a cycle, it features outgoing transitions (such as, for instance, transition $\langle a_1, b_2, c_0, d_0 \rangle \rightarrow _{U^{\mathsf {asyn}}} \langle a_0, b_2, c_0, d_0 \rangle$) and thus is not a trap domain.

The aim of the rest of this paper is to tackle the enumeration of fixed points ("Fixed points enumeration") and attractors ("Length* n* attractors enumeration") in an AN. For this, we use ASP ("Answer set programming") which is a declarative paradigm dedicated to the resolution of complex problems.

## Answer set programming

In this section, we briefly recapitulate the basic elements of ASP [18], a declarative language that proved efficient to address highly computational problems. An answer set program is a finite set of rules of the form:

1 $$\begin{aligned} a_{0}\ \leftarrow \ a_{1},\ \ldots ,\ a_{m},\ not\ a_{m+1},\ \ldots ,\ not\ a_{n}. \end{aligned}$$

where $n \ge m \ge 0$, $a_{0}$ is an atom or $\bot$, all $a_{1}, \ldots ,a_{n}$ are atoms, and the symbol “*not*” denotes negation as failure. The intuitive reading of such a rule is that whenever $a_{1}, \ldots , a_{m}$ are known to be true and there is no evidence for any of the negated atoms $a_{m+1}, \ldots , a_{n}$ to be true, then $a_{0}$ has to be true as well. An atom or a negated atom is also called a literal.

Some special rules are noteworthy. A rule where $m = n = 0$ is called a fact and is useful to represent data because the left-hand atom $a_0$ is thus always true. It is often written without the central arrow [see rule (2)]. On the other hand, a rule where $n > 0$ and $a_0 = \bot$ is called a constraint. As $\bot$ can never become true, if the right-hand side of a constraint is true, this invalidates the whole solution. Constraints are thus useful to filter out unwanted solutions. The symbol $\bot$ is usually omitted in a constraint [see rule (3)].

2 $$\begin{aligned}&a_{0}.\end{aligned}$$

3 $$\begin{aligned}&\leftarrow \ a_{1},\ \ldots ,\ a_{m},\ not\ a_{m+1},\ \ldots ,\ not\ a_{n}. \end{aligned}$$

In the ASP paradigm, the search of solutions consists in computing the answer sets of a given program. An answer set for a program is defined by Gelfond and Lifschitz [26] as follows. An interpretation *I* is a finite set of propositional atoms. A rule *r* as given in (1) is true under I if and only if:

$$ \{ a_{1} , \ldots ,a{}_{m}\}  \subseteq I \wedge \{ a_{{m + 1}} , \ldots ,a{}_{n}\}  \cap I = \emptyset  \Rightarrow a_{0}  \in I $$

An interpretation *I* is a model of a program *P* if each rule $r \in P$ is true under *I*. Finally, *I* is an answer set of *P* if *I* is a minimal (in terms of inclusion) model of $P^{I}$, where $P^{I}$ is defined as the program that results from *P* by deleting all rules that contain a negated atom that appears in *I*, and deleting all negated atoms from the remaining rules.

Programs can yield no answer set, one answer set, or several answer sets. For example, the following program:

4 $$\begin{aligned}&b \ \leftarrow \ not\ c. \end{aligned}$$

5 $$\begin{aligned}&c \ \leftarrow \ not\ b. \end{aligned}$$

produces two answer sets: $\{b\}$ and $\{c\}$. Indeed, the absence of *c* makes *b* true, and conversely absence of *b* makes *c* true. Cardinality constructs are another way to obtain multiple answer sets. The most usual way of using a cardinality is in place of $a_0$:

$$\begin{aligned} l \ \{q_{1}, \ \ldots , \ q_{k}\} \ u \ \leftarrow \ a_{1},\ \ldots ,\ a_{m},\ not\ a_{m+1},\ \ldots ,\ not\ a_{n}. \end{aligned}$$

where $k \ge 0$, *l* is an integer and *u* is an integer or the infinity ($\infty$). Such a cardinality means that, under the condition that the body is satisfied, the answer set *X* must contain at least *l* and at most *u* atoms from the set $\{q_{1},\ldots, q_{m}\}$, or, in other words: $l \le | \{q_{1},\ldots, q_{m}\} \cap X | \le u$ where $\cap$ is the symbol of sets intersection and |*A*| denotes the cardinality of set *A*. We note that several answer sets may match this definition, as there may be numerous solutions *X* to this equation. Using cardinalities, the program example of (4) and (5) can be summed up into the following program containing one only fact:

$$\begin{aligned} 1 \ \{b, c\} \ 1. \end{aligned}$$

If they are not explicitly given, *l* defaults to 0 and *u* defaults to $\infty$. Furthermore, if such a cardinality is found in the body of a rule, then it is true if the above condition is satisfied.

Atoms in ASP are expressed as predicates with an arity, that is, they can apply to terms (also called arguments). For instance, let us take the following program:

$$\begin{aligned}&fishesCannotFly. \\&fish(shark). \\&livesIn(X,water) \ \leftarrow \ fish(X),\ fishesCannotFly. \end{aligned}$$

The intuitive meaning of this program is that if fish do not fly (which is the case) and that something is a fish, then this thing lives in water. Here, *fishesCannotFly* is a predicate with arity zero (no terms), *fish* has arity one (one term, defining something that is a fish), and *livesIn* has arity two (the first term lives in the second term). On the other hand, the terms *shark* and *water* are constants while *X* is a variable, which can stand for any atom. By convention, constant names start with a low letter or are written in quotes, and variable names start with a capital letter.

However, solving an ASP program as explained above requires that it contains no variable; for this, a grounding step is first required, consisting in the removal of all free variables by replacing them by possible constants while preserving the meaning of the program. In the example above, the grounding step produces the following variable-free program, where *X* is replaced by the only suitable constant *shark*:

$$\begin{aligned}&fishesCannotFly. \\&fish(shark). \\&livesIn(shark,water) \ \leftarrow \ fish(shark),\ fishesCannotFly. \end{aligned}$$

After solving, the only answer set corresponding to this program is:
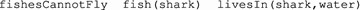


For the present work, we used Clingo 1 [27] which is a combination of a grounder and a solver. In the rest of this paper, we use ASP to tackle the problems of enumerating all fixed points and attractors of a given AN model.

## Fixed points enumeration

The first aspect of our work is the enumeration of a special type of trap domains: fixed points (also called stable states or steady states) which are composed of only one global state (see Definition 9). They can be studied separately from attractors because their enumeration follows a different pattern which is more specific to this problem. A previous version of this work using another framework (process hitting) is presented in [19]. Although the main idea is preserved, the work we present here is different because we are interested in the more expressive AN framework in which the transitions have a different form.

### Translating automata networks into answer set programs

Before any analysis of an AN, we first need to express it with ASP rules. We developed a dedicated converter named AN2ASP 2 and we detail its principle in the following.

First, the predicate automatonLevel(A,I) is used to define each automaton A along with its local state I. The local transitions are then represented with two predicates: condition which defines each element of the condition along with the origin, and target which defines the target of the local transition. Each local transition is labeled by an identifier that is the same in its condition and target predicates. Example 8 shows how an AN model is defined with these predicates.

#### *Example 8*

**(Representation of AN model in ASP)** Here is the representation of the AN model of Fig. 1 in ASP: 
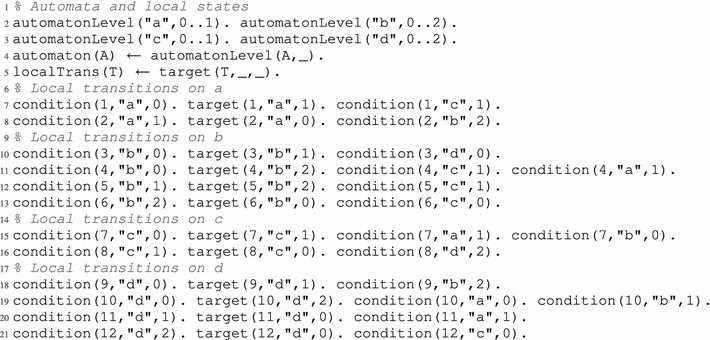


In lines 2–3 we define all the model automata with their local states. For example, the automaton “a” has two levels numbered 0 and 1; indeed, rule automatonLevel(“a”, 0..1). of line 2, for instance, will in fact expand into the two following rules:
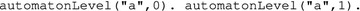


Besides, all the local transitions of the network are defined in lines 7–21; for instance, all the predicates in line 7 declare the transition $\tau _1 = a_0 \overset{\{c_1\}}{\longrightarrow } a_1$, which is labeled 1. We declare as many predicates condition as necessary in order to fully define a local transition $\tau$ that has potentially several elements in its condition $\mathsf {cond}(\tau )$. For instance, transition $b_0 \overset{\{a_1, c_1\}}{\longrightarrow } b_2$ is defined in line 11 with label 4 and requires three of these predicates for $b_0$, $a_1$ and $c_1$. Finally, in lines 4–5, predicate automaton gathers all existing automata names in the model, and predicate localTrans gathers all transition labels. The underscore symbol (_) in the parameters of a predicate is a placeholder for any value.

Since the names of the biological components may start with a capital letter, it is preferable to use the double quotes (“”) around the automata names in the parameters of all predicates to ensure that the automata names are understood as constants by the ASP grounder and not as variables.

### Fixed points search

The enumeration of fixed points requires to encode the definition of a fixed point (given in Definition 9) as an ASP program through logic rules. The first step of this process is to browse all the possible states of the network; in other words, all possible combinations of automata local states are generated by choosing exactly one local level for each automaton. However, before computing these combinations, we need to pre-process the list of the selected local states in order to exclude each local state $a_i$ such that there exists a local transition $a_i \overset{\emptyset }{\rightarrow } a_j \in \mathcal {T}$. Such local states cannot be stable, because the local transition given above, called self-transition, is always playable: $\forall \zeta \in \mathcal {S}, a_i \in \zeta \Rightarrow a_i \overset{\emptyset }{\rightarrow } a_j \in P_{\zeta }$. This process is done through lines 23–27.
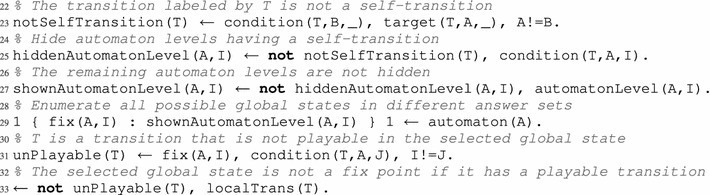


Line 29 constitutes a cardinality rule (as defined in "Answer set programming") whose consequence is the enumeration of all global states of the model in distinct answer sets. Each global state is defined by considering exactly one local state for each existing automaton from the shown ones defined in shownAutomatonLevel. Each global state is described using predicates fix(A,I), named in anticipation of the final fixed point results, where I is the active local state of automaton A.

The last step consists in filtering out any global state $\zeta$, that is not a fixed point, among all generated states. In this case, it consists in eliminating all candidate answer sets in which at least one local transition can be played, that is, where $P_\zeta \ne \emptyset$. Such a filtering part is ideally realized with the use of one or several constraints. As explained in "Answer set programming", a constraint removes all answer sets that satisfy its right-hand part. Regarding our problem, an answer set representing a given global state must be filtered out if there exists at least one playable local transition in this state (line 33). A transition T is considered as unplayable in a state, that is, $\texttt {T} \notin P_\zeta$, if at least one of its conditions is not satisfied. For this, predicate unPlayable(T) defined in line 31, flags a local transition as unplayable when one of its condition contains a local state that is different from the local state of the same automaton. This is used in the final constraint (line 33) which states that if there exists a local transition which is playable in the considered global state (i.e., $\exists \texttt {T} \in \mathcal {T}, \texttt {T} \in P_\zeta$) then this global state should be eliminated from the result answer sets (because it is not a fixed point). In the end, the fixed points of a considered model are exactly the global states represented in each remaining answer sets, described by the set of the atoms fix(A,I) which define each automaton local state.

#### *Example 9*

(Fixed point enumeration) The AN model of Fig. 1 contains 4 automata: *a* and *c* have 2 local states while *b* and *d* have 3; therefore, the whole model has $2*2*3*3 = 36$ states (whether they can be reached or not from a given initial state). We can check that this model contains exactly 3 fixed points: $\langle a_1, b_1, c_0, d_0 \rangle$, $\langle a_1, b_1, c_1, d_0 \rangle$ and $\langle a_0, b_0, c_0, d_1 \rangle$. All of them are represented in both Figs. 2 and 3. In this model, no other state verifies this property. We recall that the fixed points are identical for the synchronous and asynchronous update schemes [24].

If we execute the ASP program detailed above (lines 23–33) alongside with the the AN model given in Example 8 (lines 1–21), we obtain 3 answer sets that match the expected result. The output of Clingo is the following:
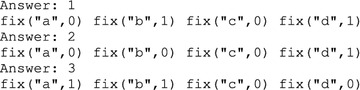


## Length *n* attractors enumeration

In the previous section we gave a method to enumerate all fixed points of a given model. In a sense, a fixed point can be considered as an attractor: it cannot be escaped and its size ($n=1$) makes it trivially minimal. However, attractors in the general case are made of several states. In the rest of this paper, we exclude one-state attractors (tackled in the last section "Fixed points enumeration"). We focus on attractors composed with several-states (following Definition 11) and we describe how to obtain some or all the attractors of a given length in a model. Obtaining all attractors of any length can be theoretically tackled by gradually increasing the considered length.

The computational method to enumerate all attractors of length n in AN models consists in three steps:

1. Enumerate all paths of length n,
2. Remove all paths that are not cycles,
3. Remove all cycles that are not trap domains (i.e., keep only attractors).

Once all steps are passed, each trace of the remaining n-length paths is an attractor (following Lemma 3).

### Cycles enumeration

The approach presented here first enumerates all the paths of length n in the AN model (Definition 7).

In an ASP program, it is possible to instantiate constants whose values are defined by the user at each execution: this is the role of the lowercase n in step(0..n) (line 26), that represents the number of considered steps. For example, knowing the initial global state, step(0..5) will compute all paths of length 5 (thus containing 6 successive global states).

In order to enumerate all the possible paths, step 0 should take the value of all the possible initial global states (line 28), in a similar way to the fixed point enumeration. Then, identifying the successors of a given global state requires to identify the set of its playable local transitions. We recall that a local transition is playable in a global state when its origin and all its conditions are active in that global state (see Definition 2). Therefore, we define an ASP predicate unPlayable(T,S) in line 30 stating that a transition T is not playable at a step S. More precisely, T cannot be played in the corresponding global state of the system at step S, which is the case when at least one of its conditions is not satisfied. Obviously, each local transition that is not unplayable, is a playable. From this, we will be able to flag the actually played local transitions with played(T,S) (see later in lines 33 and 39).
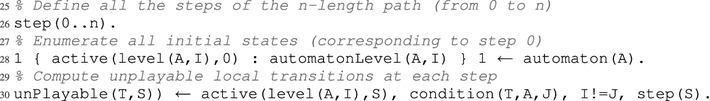


In our approach, we tackle separately the computation of the dynamics and the resolution of our problem (namely, attractors enumeration). We show in the following how to compute the evolution of the model through the asynchronous and the synchronous update schemes, as presented in "Update schemes and dynamics of automata networks". The piece of program that computes the attractors, given afterwards, is common to whatever update schemes.

All possible evolutions of the network (that is, the resulting paths after playing a set of global transitions) can be enumerated with a cardinality rule (explained in "Answer set programming") such as the ones in line 33 for the asynchronous update scheme, and line 39 for the synchronous update scheme. Such rules reproduce all possible paths in the dynamics of the model by representing each possible successor of a considered state as an answer set. This enumeration encompasses the non-deterministic behavior (in both update schemes).

To enforce the strictly asynchronous dynamics which requires that exactly one automaton changes during a global transition, we use the constraint of line 35 to remove all paths where no local transition has been played, and the constraint of line 36 to remove all paths where two or more local transitions have been played simultaneously. Thus, all the remaining paths contained in the answer sets strictly follow the asynchronous dynamics given in Definition 3. The underscore symbol (_) in the parameters of a predicate is a placeholder for any value. Here, it is used in place of the transition label, meaning that these rules are applicable to any transition. 
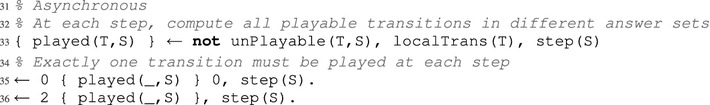


The second update scheme corresponds to synchronous dynamics in which all playable transitions that are not in-conflict have to be played (see Definition 4). Furthermore, “empty” global transition are not allowed, even when when no transition is playable (line 41).
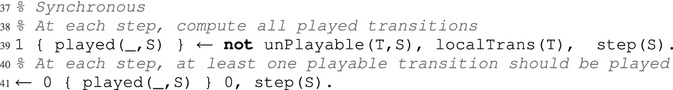


In a nutshell, one should choose one of both pieces of program presented above, that is, either lines 39–41 for the asynchronous update scheme, or lines 39–41 for the synchronous one. The overall result of both of these pieces of programs is a collection of answer sets, where each answer is a possible path of length n (that is, computed in n steps) and starting from any initial state (at step 0).

Between two consecutive steps S and S+1, we witness that the active level of a given automaton B has changed with the predicate change in line 43, which stores the chosen local transition.

In-conflict local transitions (see Definition 6) cannot be played at the same step. They are the only source of non-determinism in the synchronous update scheme, because the dynamics has to “choose” which local transition to take into account. This property is verified by the constraint in line 45, that states that at most one change can occur (i.e., one transition can be played) in the same automaton. Finally, it is necessary to compute the content of the new global state after each played global transition: if a change is witnessed, then the related automaton has to change its level into the local state of the local transition destination (lines 47–48) otherwise it remains the same (line 49).
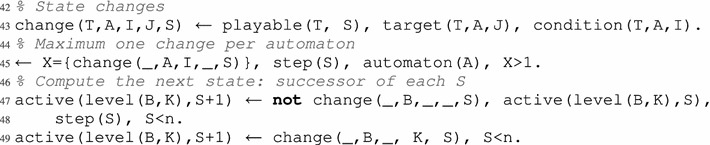


After the construction of a path of length n, it is required to check whether it is a cycle or not. If it is a cycle, then consequently it is a strongly connected component (see Lemma 2). To do so, we need a predicate different(S1,S2) (lines 52–54) which is true when an automaton has different active levels in two global states visited at steps S1 and S2. On the contrary, if different(S1,S2) is not true, this means that all active levels of all automata are the same in both states. Thus, there is a cycle between S1 and S2 (line 56). We finally eliminate all the paths that are not cycles of size n with the constraint of line 59, that checks if the states at steps 0 and n are identical.
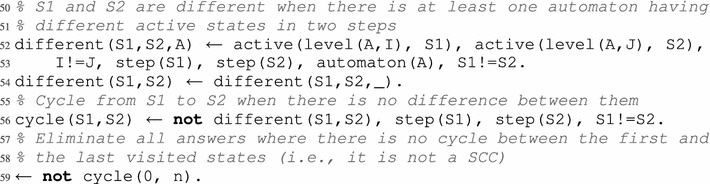


As given in Lemma 2, all remaining paths are strongly connected components. We finally need to verify if they are trap domains (Lemma 3) in order to discriminate attractors.

### Attractors enumeration

Due to the non-deterministic behavior in the dynamics, each state in the state-transition graph of a given AN may have several successors. Therefore a cyclic path is not necessarily an attractor. The only certain exception is the case of the deterministic synchronous update scheme (such as in Boolean models, as explained in Section "Determinism and non-determinism of the update schemes"). In this case, the computation may be stopped here because a cycle is necessarily an attractor. This result is used in [28–30].

In the rest of this section, we will tackle the more general and challenging case of non-determinism. Indeed, in the general case, some local transitions may allow the dynamics to escape the cycle; in such case, the cycle would not even be a trap domain (see Lemma 4). For instance, in the partial state-transition graph of Fig. 2, we can spot many cycles of various lengths but not all of them are attractors. In particular, the initial global state is part of a cycle of length 2 which is not an attractor, and which trace is: $\{ \langle a_1, b_2, c_0, d_1 \rangle , \langle a_1, b_2, c_0, d_0 \rangle \}$.

That is why another check is required to filter out all the remaining cycles that can be escaped (and are therefore not attractors). Once again, this filtering is performed with constraints, which are the most suitable solution. In order to define such constraints, we need to describe the behavior that we do not wish to observe: escaping the computed cycle. For this, it is necessary to differentiate between the effectively played local transitions (played) and the ”also playable” local transitions which were not played (alsoPlayable in line 61). Then, we verify at each step S, comprised between 0 and n, if these also playable local transitions make the system evolve or not to a new global state that is not a part of the cycle trace.

For the asynchronous update scheme, any also playable local transition can potentially make the dynamics leave the cycle. Regarding the synchronous update scheme, an also playable local transition must necessarily be in-conflict (see Definition 6) with a local transition used to find the studied cycle. Nevertheless, both cases are tackled jointly. The predicate alsoPlayable(T,S) states that a local transition T is also playable at step S in the considered cycle, but was not used to specifically build the said cycle. This predicate is similar to the predicate playable used previously in lines 30, 33 and 39.
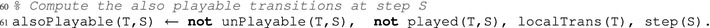


After finding these also playable local transitions in each state of the cycle, we have to verify if all its global states, found by applying these also playable local transitions, are as well part of the cycle. Indeed, it is possible to have an also playable local transition that makes the dynamics evolve inside the cycle; this is witnessed by the predicate evolveInCycle (lines 64–65). Such transitions are simply “shortcuts” to other states in the same cycle. This is the case in complex attractors, that do not simply consist in a single cycle but are made of a composition of cycles. Such global transitions are disregarded in the current case as we are only interested in finding global transitions that would allow the model dynamic to escape from the cycle. Instead, we are interested in filtering out cases where a transition allows to exit the cycle (that is, leads to a state not featured in the trace of the cycle) using the constraint of line 68.
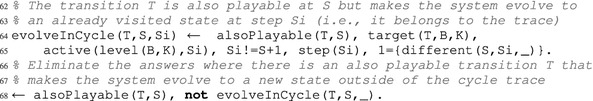


#### *Example 10*

In the dynamics of the networks presented in Fig. 1 with the asynchronous update scheme, let us consider the following cycle of length 2, which can be seen in Fig. 2: $\langle a_1, b_2, c_0, d_1 \rangle \rightarrow _{U^{\mathsf {asyn}}} \langle a_1, b_2, c_0, d_0 \rangle \rightarrow _{U^{\mathsf {asyn}}} \langle a_1, b_2, c_0, d_1 \rangle$. Following the pieces of program given in this section, one of the answer sets could allow to find this cycle, among others, by returning in particular the following predicates:
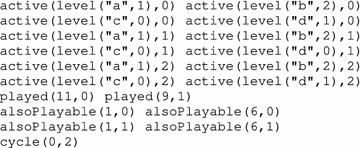
 The three states in the cycle are labeled 0, 1 and 2, and the active local states they contain are described by the predicate active. We note that states 0 and 2 are identical, which is witnessed by the atom cycle(0,2). Furthermore, predicate played give the two transitions (labeled 9 and 11, see lines 18 and 20) allowing to run through all the states of the cycle, while predicate alsoPlayable give the local transitions that are “also playable” in the cycle; indeed, in both states, the transitions labeled 1 and 6 are playable. Finally, no evolveInCycle predicate is inferred for this example (the only also playable transition is 1 which makes the dynamics evolve outside the cycle). Thus, this answer set is discarded thanks to the constraint of line 68 and is not featured among the results.

### Complex attractors

Up to this point, we managed to propose an ASP program that enumerates all the attractors in a given AN. Each attractor is the trace of a path of length n. In many cases, except for some complex attractors, this length n (which corresponds to the number of played global transitions in the path) is also equal to the number of visited states (i.e., the size of the trace). This is a trivial case of a minimal path covering a given attractor, that is, no path of lesser length can cover it. Indeed, as in the examples of attractors in Figs. 2 and 3, enumerating the paths of length 2 is enough to obtain all the attractors having two global states, and the same goes for the attractors of length 4. But without the constraint that we develop below (given in lines 70–93), when the program is asked to display the attractors covered by a path of length n, it will also return various paths of size lower than n by considering non-minimal paths, that is, containing unwanted repetitions inside the cycle, or even repetitions of the entire cycle. In the example of Fig. 3, for instance, with $\texttt {n}=6$, the program returns the two attractors, although they both are of size 2. Indeed, each of them can be covered by a cycle of length 6: it consists of a cycle of size 2 repeated three times.

Therefore, the objective of this section is to exclude most cases where a cycle is non-minimal, such as the obvious one where it is entirely repeated, because such a case is useless with respect to the computation of attractors. Moreover, we would prefer that our method yields no answer set when no attractor traversed by a cycle of length n is found (even if non-minimal attractors on cycles of lesser length are found). We don’t formally claim here that our method eliminates all of these cases, but we aim at tackling most of these cases in order to sanitize the answer set as much as possible. For instance, an attractor $\zeta _0 \rightarrow \zeta _1 \rightarrow \zeta _0$ of length $\texttt {n}=2$ could be listed among the attractors of length $\texttt {n}=4$ if it is repeated twice as the following path: $\zeta _0 \rightarrow \zeta _1 \rightarrow \zeta _0 \rightarrow \zeta _1 \rightarrow \zeta _0$. Although all general assumptions regarding attractors are verified (it consists in a cycle and all the global transitions produce global states that are still cycle), we aim at willingly excluding it from the answers because it is not minimal in terms of length.

**Fig. 4:**
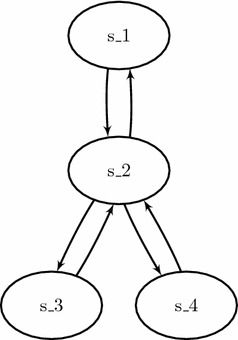
Simple example featuring a “star attractor”, that is, an attractor that cannot be traveled without visiting at least twice one of its states

However, in the case of some complex attractors, the problem is opposite. Indeed, it happens that the dynamics has to visit the same global states more than once. It is for example the case for the complex attractor which could be called “star attractor”, which is featured in the model comprising the following global transitions, also depicted in Fig. 4: $\{ \zeta _0 \rightarrow \zeta _1, \zeta _1 \rightarrow \zeta _0, \zeta _1 \rightarrow \zeta _2, \zeta _1 \rightarrow \zeta _3, \zeta _2 \rightarrow \zeta _1, \zeta _3 \rightarrow \zeta _1 \}$. The only attractor of this model consists in the whole set $\mathcal {S}= \{ \zeta _0, \zeta _1, \zeta _2, \zeta _3 \}$ of all its global states. We notice that it is not possible to cover this entire attractor without visiting the state $\zeta _1$ at least 3 times (even when disregarding the inevitably repeated final step of the cycle). Indeed, a possible path to cover it entirely is: $\zeta _0 \rightarrow \zeta _1 \rightarrow \zeta _2 \rightarrow \zeta _1 \rightarrow \zeta _3 \rightarrow \zeta _1 \rightarrow \zeta _0$ which is of length 6, and no path of lesser length exist to cover this attractor although its trace is of size 4.

The challenge here is to handle both cases in the same program: excluding answer sets featuring non-minimal paths while still returning complex attractors for which the path is strictly bigger than the trace. For this, we make direct use of the result of Lemma 1 which links the length n of a path to the size X of its trace; in our case: X = n + 1 - k, where k is the number of global states that are successively repeated in the path of length n (see Definition 8). This formula is implemented in lines 70–76. It is also used to prompt the user with the size of the attractor which may be strictly inferior to the value of n.
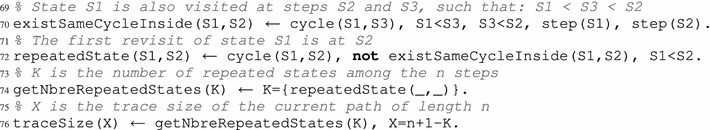


Our objective in the following is to propose a program that returns, as far as possible, all attractors of the model that actually correspond to a path of length n which is minimal. We propose the following rules to verify this property; each of them concludes with the atom isNotMinimal(n), which means that the considered cycle is not minimal. In the end, isNotMinimal(n) is used in the constraint of line 93 which eliminates all these unwanted cases together.

We first verify if there exists a path of length X < n without repetitions from the state of step 0 to step X, where X is the trace size of the cycle, that is, the number of different states in the path. Then we also verify if there is a transition from the state of step X to the state of step 0. If both properties are true, then there exists a path of size X < n that covers all the states of the attractor, and thus n is not the minimal path length of that attractor (lines 81–84).

Another non-minimal case, detailed in lines 86–87, occurs when there exists “shortcuts” between some states of a cycle, making it not minimal. Besides, a path of minimal length does not permit repetitions between successive states inside a cycle (line 89). Finally, when an entire cycle is repeated several times, then the number of repetitions is obviously superior to the maximum expected that is equal to n (line 91). As stated before, in any of the previous cases, the considered cycle is not minimal, and therefore discarded (line 93).
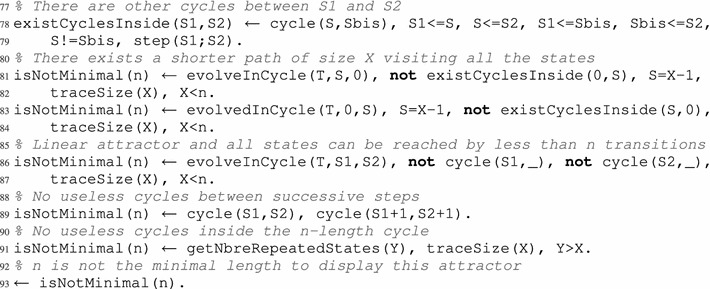
 We note that these constraints are relevant to the non-deterministic dynamics, whether it is asynchronous or synchronous.

Nevertheless, there is still a case of duplicate results that cannot be tackled by the previous constraint: the existence of several minimal cycles for the same attractor. Indeed, for one given attractor, it is possible to find several minimal covering cycles by changing the initial state, or the traversal (in the case of complex attractors). For instance, the hypothetical attractor $\{ \zeta _0 ; \zeta _1 \}$ is captured by the two cycles: $\zeta _0 \rightarrow \zeta _1 \rightarrow \zeta _0$ and $\zeta _1 \rightarrow \zeta _0 \rightarrow \zeta _1$. This leads to repetitions which are not removed from the answers of our method.

The final result presented by each answer set is described by the collection of atoms active(ALs,S), where S denotes the label of one of the steps in the cycle, and ALs corresponds to one of the active local states.

The problem of finding attractors in a discrete network is NP-hard, therefore the implementation that we gave in this section also faces such a complexity. However, ASP solvers (namely, Clingo in our case) are specialized in tackling such complex problems. Next section will be dedicated to the results of several computational experiments that we performed on biological networks. We show that our ASP implementation can return results in only a few seconds attractors of small size even on models with 100 components, which is considered large.

## Results

In this section, we exhibit several experiments conducted on biological networks. We first detail the results of our programs on the AN model of Fig. 1. Then, we sum up the results of benchmarks performed on other models up to 100 components. In general, the time performances are good and the overall results confirm the applicability ASP for the verification of formal properties or the enumeration of special constructs on biological systems.

All experiments were run on a desktop PC with a Pentium VII 3 GHz processor and 16 GB memory.

### Case study

We first conducted detailed experiments on the 4-components model of Fig. 1. As detailed in "Automata networks", this network contains 4 automata and 12 local transitions. Its state-transition graph comprises 36 different global states and some of them are detailed in the partial state-transition graphs in Fig. 2 (for the asynchronous update scheme) and Fig. 3 (for the synchronous update scheme).

The analytic study of the minimal trap domains on this small network allows to find the following attractors and fixed points depending on the update scheme, where we assimilate steady states to attractors of length $\texttt {n} = 0$ because they have a trace of size 1:

- Asynchronous update scheme:
  - $$\texttt {n}=0$$: $$\langle a_1, b_1, c_1, d_0 \rangle $$,$$\langle a_1, b_1, c_0, d_0 \rangle $$ and $$\langle a_0, b_0, c_0, d_1 \rangle $$;
  - $$\texttt {n}=2$$: $$\{ \langle a_0, b_1, c_0, d_0 \rangle , \langle a_0, b_1, c_0, d_2 \rangle \}$$;
  - $$\texttt {n}=4$$: $$\{ \langle a_1, b_2, c_1, d_1 \rangle , \langle a_0, b_2, c_1, d_1 \rangle , \langle a_0, b_2, c_1, d_0 \rangle , \langle a_1, b_2, c_1, d_0 \rangle \}$$.
- Synchronous update scheme:
  - $$\texttt {n}=0$$: $$\langle a_1, b_1, c_1, d_0 \rangle $$,$$\langle a_1, b_1, c_0, d_0 \rangle $$ and $$\langle a_0, b_0, c_0, d_1 \rangle $$;
  - $$\texttt {n}=2$$: $$\{\langle a_0, b_1, c_0, d_0 \rangle , \langle a_0, b_1, c_0, d_2 \rangle \}$$ and $$\{ \langle a_1, b_2, c_1, d_1 \rangle , \langle a_0, b_2, c_1, d_0 \rangle \}$$.

The steady states returned by the method of "Fixed points enumeration" ($\texttt {n} = 0$) and the attractors ($\texttt {n} > 1$) given by the method of "Length* n* attractors enumeration" are consistent with what is theoretically expected. We note that, as stated in [24], the fixed points are the same for the asynchronous and synchronous update schemes.

When given to a solver, the ASP programs given in the previous sections directly outputs the expected solutions. The output for the fixed point enumeration was given in Example 9. The output for the attractor enumeration is given below for both update schemes. We note that each global that state belongs to an attractor is labeled with a number (for instance, 0 and 1 for the cases $\texttt {n}=2$) so that each active local state is featured in an independent atom. We removed some uninteresting atoms from the results to improve readability.
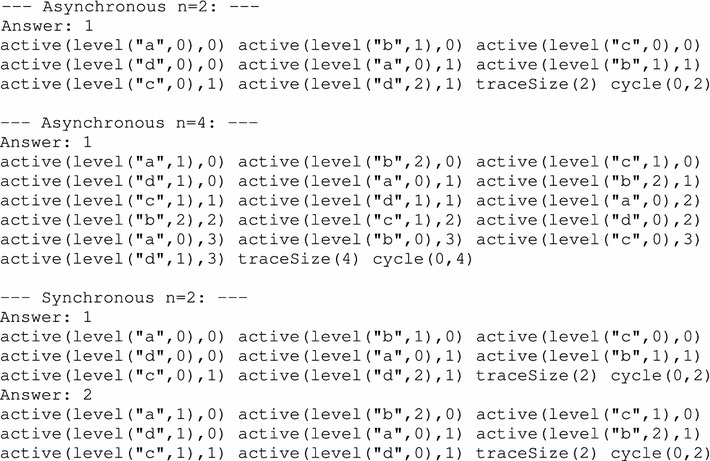


Moreover, executing the programs with $\texttt {n}\ne 2$ and $\texttt {n}\ne 4$ returns no results, which means that the solver correctly terminates having found no answer set. This is expected because there is no attractor of length different than 2 and 4 for this model, and we excluded repeated cycles from the results (therefore, the attractors already found for lengths 2 and 4 are not found for $\texttt {n}=6$ or $\texttt {n}=8$, for instance). For this small network, all the results are computed in less than 0.05 second.

### Benchmarks

In the following, we propose some additional benchmarks to demonstrate the capabilities of our implementation. We do not give the details of the results of these experiments but rather focus on the computation times and the conclusion: if an attractor has been found (satisfiable) or not (unsatisfiable). We used several preexisting Boolean and multi-valued networks inspired from real organisms and found in the literature:

- **Lambda phage:** a regulatory network featuring some viral genes crucial in the decision between lysis and lysogenization in temperate bacteriophage lambda [31];
- **Trp-reg:** a qualitative model of regulated metabolic pathways of the tryptophan biosynthesis in* E. coli* [32];
- **Fission-yeast:** a cell cycle model of* Schizosaccharomyces pombe* [33];
- **Mamm:** a mammalian cell cycle model [34];
- **Tcrsig:** a signaling and regulatory network of the TCR signaling pathway in the mammalian differentiation [35];
- **FGF:** a drosophila FGF signaling pathway [36];
- **T-helper:** a model of the T-helper cells differentiation and plasticity, which accounts for novel cellular subtypes [37].

To obtain the models that we have studied in this section, we first extracted them from the GINsim model repository^3^ [38], in GINML format. These models correspond to the discrete asynchronous networks given in the corresponding papers. Then, the conversion step towards an ASP program is automated using the following tools:

- The existing GINsim tool allows to export its models into the *SBML qual* formalism;
- The existing LogicalModel library [39, 40] can convert *SBML qual* models into AN models;
- Finally, our script AN2ASP converts AN models into ASP programs, following the principles detailed in "Translating automata networks into answer set programs".

It is noteworthy that each step fully preserves the dynamics between models regarding the asynchronous update scheme [41]; thus, the final (asynchronous) ASP program is bisimilar to the original GINML model. The characteristics of each model once translated in AN are given in Table 1. The results of our benchmarks^4^ are given in Tables 2 and 3.

**Table 1:** Brief description of the models used in our benchmarks

Models		Model description			
		$|\Sigma |$	$\max _{a\in \Sigma }\{|\mathcal {S}_a|\}$	$|\mathcal {T}|$	$|\mathcal {S}|$
	Example	4	3	12	36
[31]	Lambda phage	4	4	46	48
[32]	Trp-reg	4	3	14	36
[33]	Fission-yeast	9	3	43	3 $\times$ 2$^\text {9}$ $=$ 1536
[34]	Mamm.	10	2	34	2$^{10}$ $=$ 1024
[35]	Tcrsig	40	2	85	2$^\text {40}$ $\simeq$ 10$^{12}$
[36]	FGF	59	3	102	2$^\text {31}$ $\simeq$ 1.2 $\times$ 10$^\text {10}$
[37]	T-helper	101	3	316	2$^\text {102}$ $\simeq$ 5.7 $\times$ 10$^\text {31}$

The successive lines sum up the information regarding the models of, respectively, the toy example of Fig. 1, the bacteriophage lambda [31], the tryptophan biosynthesis in* E.coli* regression [32], the fission yeast [33], the mammilian cell cycle [34], the TCR signalling pathway in the mammalian differentiation [35], the drosophila FGF signalling pathway [36] and the T-helper cell differentiation [37]. For each of them, the table gives the number of automata ($|\Sigma |$), the maximal local level in the automata ($\max _{a\in \Sigma }\{|\mathcal {S}_a|\}$), the number of local transitions ($|\mathcal {T}|$) and the number of states in the corresponding state-transition graph ($|\mathcal {S}|$)

**Table 2:** Results of our fixed points enumeration implementation

Models	Fixed points enumeration for both update schemes	
	$\Delta ^{\mathsf {all}} t$ (ms)	#$^{\mathsf {all}} \mathbf {F}$
Example	2	3
Lambda phage	4	1
Trp-reg	6	2
Fission-yeast	5	1
Mamm.	3	1
Tcrsig	5	8
FGF	25	1536
T-helper	170,642	5,875,504

The successive lines sum up the information regarding models detailed in Table 1. For each model, the table shows the computation time for the enumeration of all results and the total number of returned answer sets

We note that all the results for the fixed points search have been compared and confirmed using GINsim [38] and Pint [39]. Regarding the attractor enumeration, we compared our results with Boolean network system (BNS) [16] for the synchronous update scheme on the Fission-yeast, Mamm., and Tcrsig models; and with GINsim [38] for the asynchronous update scheme on the Lambda phage, Trp-reg, Fission-yeast and Mamm. models. In all cases, we found the same results. It is interesting to note that our method allows to return a response regarding attractors of small size even on big models. In contrast, other tools may take a very long time or even fail to answer. For instance, that happens with GINsim for the Tcrsig, FGF and T-helper models. Indeed, they are based on the computation of the complete transition graph even for the study of small attractors.

Our results could not be compared with, for example, the existing ASP-G method [17]. Indeed, with this tool, the user has to choose an update rule on which the dynamic evolution will be based on. For instance, one rule consists in activating a gene when at least one of its activators is active while no inhibitor is; another one activates a gene when it has more expressed activators than inhibitors. Because the chosen activation rule is applied for all the components of the model, while the evolution rules in our AN semantics are specific to each component, the results of both tools cannot be strictly compared.

We recall that among the results output, some attractors may be listed several times in the answers, despite any filtering, as explained at the end of "Complex attractors". Indeed, the solver returns different answer sets for different paths that cover the same trace but differ in terms of initial global state. Therefore, in the results of Table 3, we focused on the conclusion and computation times of the search of any first found attractor of length n.

In case the user may need the exhaustive list of all attractors, our method can also list all the answers, including these repetitions. For instance, our method yields 4 answers for the Trp-reg model and a cycle length of $\texttt {n} = 4$ with the asynchronous update scheme, and the computation takes 47 ms; this typically represents an attractor of size 4 where each answer set represents a cycle starting from a different initial state. Regarding the T-helper model (the largest studied model with 101 automata), the search for all attractors of size $\texttt {n} = 2$ with the synchronous update scheme takes about 275 s ($\sim$5 min) and returns 2,058,272 answers, while it takes only 57 s to return all the attractors of size n=12, (6144 answers). However, as explained before, these results mean that this model features strictly less than, for instance, 6144 attractors covered by a cycle of length 12, because each one is repeated several times.

In order to filter out the remaining repetitions, it should be possible to use a script or a text editor in order to extract only the states of each answer set and thus discard the answers featuring exactly the same attractor. Such pruning is not trivial in ASP and is the target of future works.

**Table 3:** Results of our attractors enumeration implementation

Models	n	Attractors enumeration			
		Asynchronous scheme		Synchronous scheme	
		$\Delta t$ (ms)	$\exists ?\mathbf {A}$	$\Delta t$ (ms)	$\exists ?\mathbf {A}$
Example	2	7	Yes	7	Yes
	4	16	Yes	14	No
	8	98	No	75	No
Lambda phage	2	14	Yes	14	Yes
	10	1352	No	842	No
	20	15,656	No	14,452	No
Trp-reg	2	8	No	7	No
	4	14	Yes	15	No
	20	3908	No	3808	No
Fission- yeast	2	16	No	16	Yes
	10	1011	No	807	No
	20	17,302	No	16,313	No
Mamm.	2	12	No	12	No
	7	177	No	147	Yes
	10	720	No	605	No
	20	58,133	No	9253	No
Tcrsig	2	26	No	25	No
	6	353	No	288	Yes
	10	2420	No	1841	No
	20	85,599	No	27,078	No
FGF	2	38	No	36	No
	10	2080	No	1953	No
	20	30,861	No	29,838	No
T-helper	2	180	No	125	Yes
	3	391	No	301	Yes
	4	782	No	1064	No
	6	4271	No	2372	Yes
	7	7909	No	3522	Yes
	9	26,443	No	7042	Yes
	10	44,924	No	12,208	Yes
	12	107,358	No	28,520	Yes
	20	4,230,836 $\sim$ 1h17	No	187,105 $\sim$ 3min	No

The successive lines sum up the information regarding models detailed in Table 1. For each model and for both update schemes (asynchronous and synchronous), the table shows, depending on the given path length n, the computation time for the first attractor found by the solver ($\Delta t$), and the conclusion regarding the existence or not of at least one attractor ($\exists ?\mathbf {A}$)

## Conclusion and future direction

In this paper, we presented a new logical approach to efficiently compute the list of all fixed points and attractors in biological regulatory networks. We formalized our approach using the AN framework, which is bisimilar to many logical networks [41]. All results given here can thus be applied to the widespread Thomas’ modeling [42] in the asynchronous scheme and to the Kauffman modeling in the synchronous scheme [43]. In addition, this framework can encompass any update rules, such as the ones represented in [44, 45].

We designed a dedicated method for computing steady states and other programs for non-unitary attractors of a given length and a chosen update scheme (synchronous or asynchronous). The originality of our work consists in the exhaustive enumeration of all attractors thanks to the use of ASP, a powerful declarative programming paradigm. The computational framework is based on the AN formalism presuming non-deterministic dynamics. Thanks to the encoding we introduced, and the powerful heuristics developed in modern solvers, we are able to tackle the enumeration of fixed points, cycles and attractors of large models. The major benefit of a such method is to get an exhaustive enumeration of all potential states while still being tractable for models with a hundred of interacting components. As the identification of attractors can give an insight to the long-term behavior of biological systems, tackling this issue is a challenge to which we cared to contribute to. Besides, we hope our work helps open new ways and tools to explore this field.

We plan to extend this work by considering adaptations and optimizations of the approach to address larger models. First, the “projection” feature of Clingo which displays only one answer set when several answer sets contain common predicates, is currently studied in order to filter out repeated attractors, that currently appear multiple times because they are covered by several possible cycles. Another trail consists in returning approximations of the results, that is, sometimes “missing” some answers, but with the benefit of a highly improved performance. Once again, applying various filters to the generated results may avoid redundancy and guide the solving process. Conversely, it may be possible to reduce the incremental aspect of the analysis process, for instance by searching for cycles of size lower than (and not only equal to) a given value, so that the user could directly start with higher values.

Of course, other extensions allowing to tackle other close problems would be of interest. For instance, the attractor inverse problem consists in building or enumerating networks possessing a given set of attractor properties, in order to answer to network inference matters. We would also like to extend these ASP-based methods to study other interesting properties of dynamical patterns such as the enumeration of basins of attraction, gardens of Eden or bifurcations [46].

## Footnotes

1 We used Clingo version 5.0: http://potassco.sourceforge.net/.

2 All programs and benchmarks are available as additional files and at http://www.irccyn.ec-nantes.fr/~benabdal/attractors.zip.

3
http://ginsim.org/models_repository.

4 All programs and benchmarks are available as additional files and at http://www.irccyn.ec-nantes.fr/~benabdal/attractors.zip.
